# Supplementary material for: TASI: A software tool for spatial-temporal quantification of tumor spheroid dynamics
Source: Sci Rep. 2018 May 8;8:7248. doi: 10.1038/s41598-018-25337-4 (PMC5940855; doi:10.1038/s41598-018-25337-4)
Supplement: Supplementary file 1 — Supplementary Information [file 41598_2018_25337_MOESM1_ESM.docx]

**TASI: A software tool for spatial-temporal quantification of tumor spheroid dynamics**

Yue Hou^1^, Jessica Konen^2^, Daniel J Brat^3^, Adam I Marcus^4,5^, Lee AD Cooper^1,4,6*^

^1^Department of Biomedical Informatics, Emory University, Atlanta, GA

^2^Graduate Program in Cancer Biology, Emory University, Atlanta, GA

^3^Department of Pathology and Laboratory Medicine, Emory University, Atlanta, GA

^4^Winship Cancer Institute, Emory University, Atlanta, GA

^5^Department of Hematology and Medical Oncology, Emory University, Atlanta, GA

^6^Department of Biomedical Engineering, Emory University / Georgia Tech, Atlanta, GA

* Correspondence: [lee.cooper@emory.edu](mailto:lee.cooper@emory.edu)

**SUPPLEMENTARY INFORMATION**

**SUPPLEMENTARY INFORMATION**

**SUPPLEMENTARY METHODS**

## **Cell lines and transfections**

H1299 human NSCLC cells (ATCC, Manassas, VA) were cultured in Roswell Park Memorial Institute (RPMI-1640) media supplemented with 10% fetal bovine serum and 100 units/mL of penicillin/streptomycin, and maintained at 37°C and 5% CO_2_.

The gd2PAL-Dendra2 plasmid was obtained from the Gary Bassell lab (Emory University) and was stably transfected into H1299 cells using LT-1 transfection reagent (Mirus). Geneticin was used to select for Dendra2-expressing cells at 300 μg/ml concentration (H1299 cells) or at 400 μg/ml (H1792 cells).

## **Isolation of cell phenotypes using SaGA**

H1299-Dendra2 cells were plated for spheroids, embedded in Matrigel (BD Biosciences, 2.0mg/ml), and allowed to invade overnight as described above. After about 16 hours of invasion time, spheroid plates were imaged using the Nikon A1R live cell laser scanning confocal. Spheroids were imaged using the 10x objective (0.3 NA DIC) and photoconversion was performed at a 3X zoom using the A1R software. The 405nm laser was used to photoconvert cells of interest at laser power 10-30%. A final red intensity of >300 a.f.u. was the optimal photoconversion intensity. After photoconversion, the Matrigel matrix was degraded using either trypsin at 0.125% or dispase I at 0.6ug/ml with intermittent manual disruption via pipetting. This process also broke the spheroids up into single cells. The protease activity was inactivated using media and samples were centrifuged. Upon resuspension, the samples were analyzed via FACS for TexasRed and FITC expression. Photoconverted cells were seen as a double positive population and sorted. For purified leader or follower cell collections, 30-50 cells were sorted per well and expanded.

## **Spheroid formation and invasion assays**

To generate spheroids, H1299 cells were added to a Spheron Nunclon 96 well plate (Thermo Scientific, Waltham, MA) at a concentration of 1.5 x 10^4^ cells/ml. After 2-3 days of incubation at 37^o^C, compacted spheroids were collected and resuspended in 2.0mg/ml Matrigel (BD Biosciences). Spheroids were plated in a 35 mm glass bottom dish (In Vitro Scientific) and incubated at 37^o^C overnight to allow for invasion. Images were taken at 0 and 20-24 hours post-embedding using an Olympus IX51 microscope.

## **Live spheroid confocal Imaging**

H1299 spheroids were embedded within a 2.0ug/ml Matrigel matrix and imaged using a Perkin Elmer spinning disk confocal microscope at 10x (Plan-Neofluar 0.30 NA) mounted onto a Zeiss Axiovert encased at 37^o^C with 5% CO_2_. Transmitted light images were acquired every 10 minutes for 20 hours using a Hamamatsu Orca ER CCD camera with 2X2 binning. H1299 leader and follower purified spheroids were imaged using a Leica SP8 inverted confocal microscope with live cell chamber at 10x (HC Plan Fluotar 0.3 NA). Images were collected every 10 minutes using a 488 nm argon laser, beginning 6 hours post-embedding for a total of 12 hours.

**Software**

All algorithms were developed using Matlab version 2014b. TASI utilizes the Image Processing and Statistics Toolboxes. Images are read using the ‘imread’ function which supports a variety of standard input formats (<https://www.mathworks.com/help/matlab/ref/imread.html>). Complete documentation of TASI is available at <https://github.com/cooperlab/TASI/blob/master/Instructions.pdf>.

**SUPPLEMENTARY FIGURES**


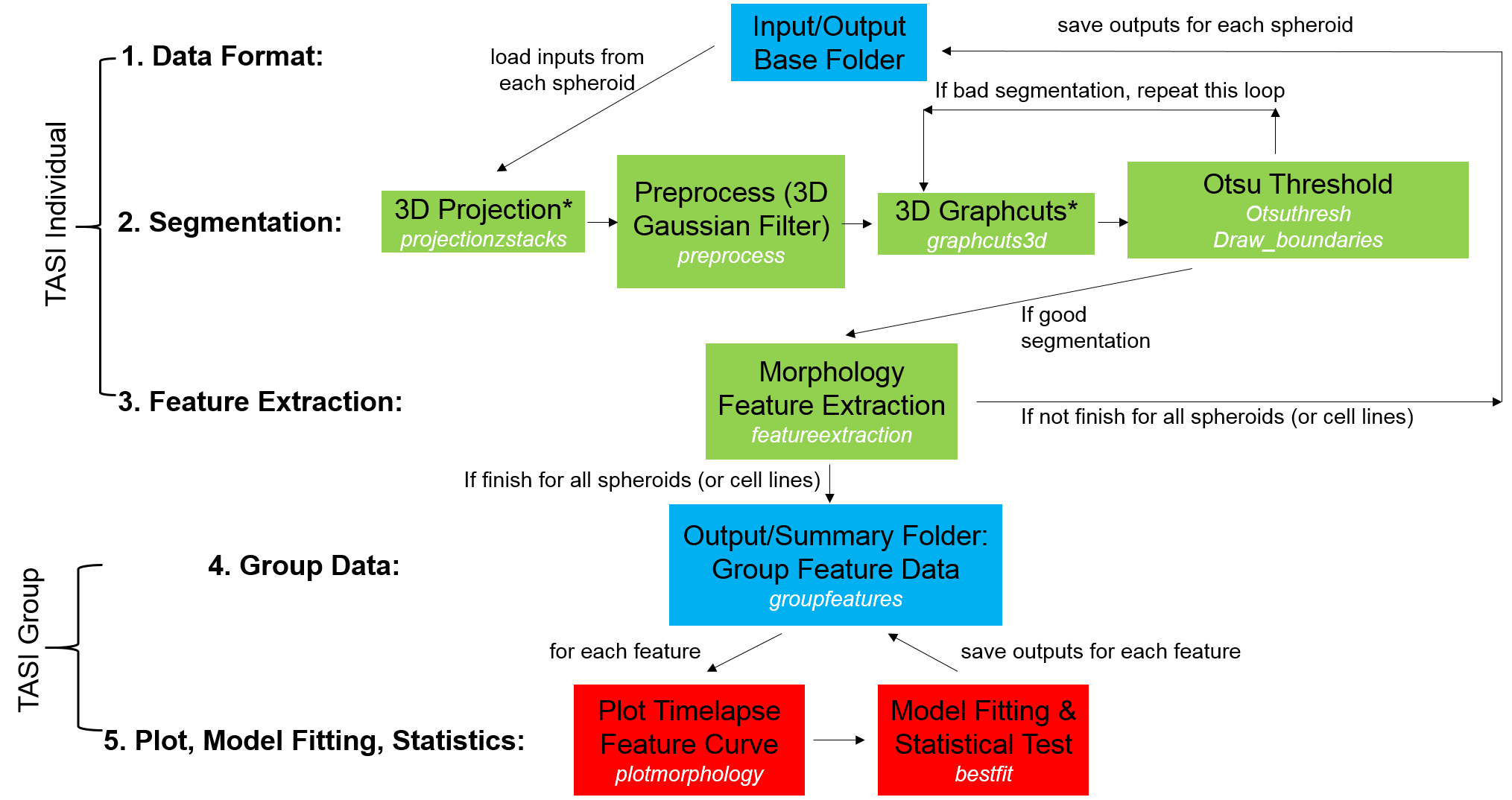


**Figure S1. Algorithm workflow.** The algorithm has two parts: TASI individual and TASI group. Each part has several steps as listed by numbers. Each step contains several functions as shown in colored boxes. The blue boxes represent the input and output folder set up. The green boxes represent the basic functions. The red boxes indicate novel functions. The functions cited from external sources are shown in the boxes with *. Arrows represent the executive sequence of each function.


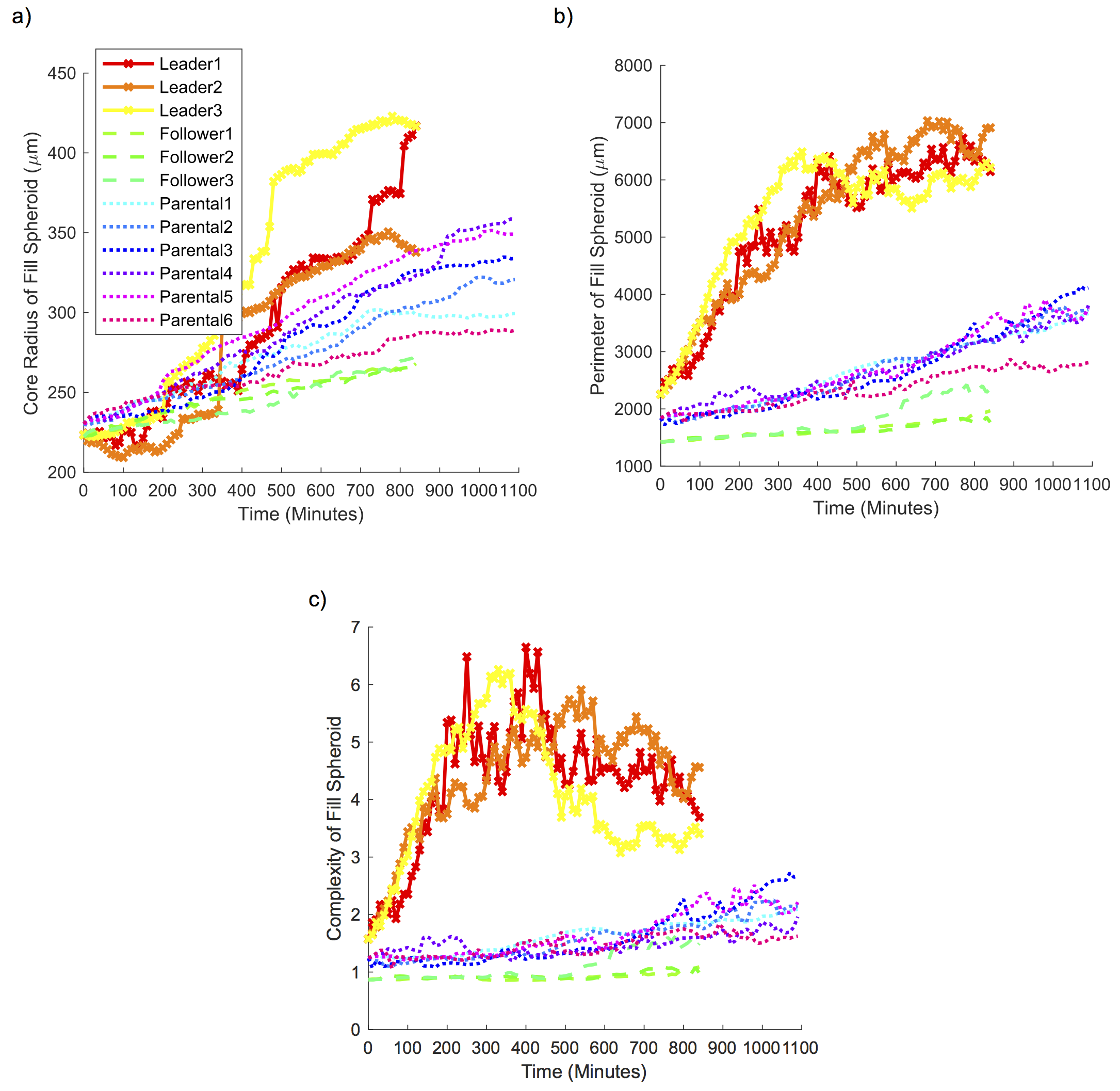


**Figure S2. Visualization of individual spheroid dynamic features.** Dynamic responses for a) core radius, b) perimeter and c) complexity for all individual spheroid. Red, orange and yellow solid lines with x symbols represent features for leader 1, 2, 3 spheroids, respectively. Light green, green and dark green dash lines represent features for follower 1, 2, 3 spheroids, respectively. Light blue, blue, dark blue, purple, pink and magenta dot lines represent features for parental 1, 2, 3, 4, 5, 6 spheroids, respectively.
